# In vitro and in vivo susceptibility of *Salmonella* spp. isolated from broiler chickens

**DOI:** 10.1007/s00580-012-1527-1

**Published:** 2012-06-15

**Authors:** Naser Ranjbar Malidareh, Sobhan Firouzi, Neda Ranjbar Malidareh, Hassan Habibi

**Affiliations:** 1 Clinician of Veterinary Diagnostic Laboratory, Babol, Iran; 2 Avian Diseases Research Center, School of Veterinary Medicine, Shiraz University, Shiraz, Iran; 3 Expert of Veterinary Diagnostic Laboratory, Babol, Iran

**Keywords:** *Salmonella*, Antimicrobial resistance, In vivo, Broiler chicken, Iran

## Abstract

Salmonellosis is the most important zoonotic disease, causing diarrhea and systemic infections. Due to poor management in antibiotic consumption, microbial resistance has increased in the treatment of zoonotic diseases. This study was conducted to evaluate the antimicrobial susceptibility of *Salmonella* spp. isolated from day-old broiler chickens which were referred to a private laboratory in Mazandaran—a province in the north of Iran—from 2008 to 2010. After harvesting the samples from the yolk sac, liver, and intestine of chickens, intestinal samples were transferred to selenite F and then incubated at 43 °C for 12–16 h. A loopful from selenite F and samples of liver and yolk sac were streaked on XLD and S.S agars. After incubation, the suspected colonies were inoculated into TSI agar for biochemical confirmation. The disk diffusion method on Muller Hinton agar was used to determine the susceptibility to antimicrobial agents. Because of the predominant use of enrofloxacin, sulfadiazine + trimethoprim, and flumequine for controlling *Salmonella* and *Escherichia coli* infections in the first week of broilers brooding in Iran, these three antibiotics were used in the in vivo study. From day 2 and continuing for 4 days, antibiotics were administrated in water, and after 10 days, samples from the liver, heart, and intestine were taken for isolation of *Salmonella*. In antimicrobial resistant tests, the most susceptible antibiotics were chloramphenicol, cefotaxime, and sulfadiazine + trimethoprim. The antimicrobial resistance to enrofloxacin, flumequine, colistin, and neomycin were 6.6, 11.6, 21.6, and 33.3 %, respectively. The results showed that 12 parties of broiler chickens were infected with paratyphoid salmonellae and the in vivo study showed that enrofloxacin and sulfadiazine + trimethoprim had the best and the weakest performance, respectively.

## Introduction

*Salmonella* spp. is among the most important food-borne pathogens in the world. Poultry and poultry products are usually incriminated in human salmonellosis outbreaks. Between 1997 and 1998, 37,842 cases of human salmonellosis were reported to the Centers for Disease Control and Prevention. The estimated number of human *Salmonella* infections in the USA exceeds 1.4 million annually (Mead et al. 1999).

*Salmonella* spp. are among the major bacterial pathogens of poultry worldwide, and most *Salmonella* infections in human result from the ingestion of contaminated poultry (Carli et al. 2001). During the last decade, there has been an alarming increase in the appearance of antibiotic-resistant bacteria as a result of poor management in antibiotic consumption. The administration of antimicrobial agents in chickens creates selection pressure that favors the survival of antibiotic-resistant pathogens. Resistance of *Salmonella* to commonly used antimicrobials is increasing, both in the veterinary field and the public health sector and has emerged as a global challenge (Molla et al. 2003). Recent studies from different countries reveal that *Salmonella* serotypes isolated from foods of animal origin have multidrug resistance profiles (Prats et al. 2000; Winokur et al. 2000; Holt et al. 2007). The aim of the present study was to determine the antimicrobial susceptibility of *Salmonella* spp. isolated from day-old broiler chickens. The efficacy of some antibiotics that are commonly used in the first week of brooding was also investigated in the experimental study.

## Materials and methods

### Samples collection

A cross-sectional study was conducted by analyzing samples of day-old broiler chickens that had referred to a private laboratory in Mazandaran, a province in the north of Iran, between 2008 and 2010. Chickens were euthanized and samples from the liver, yolk sac, and intestine were collected.

### Isolation

Samples which were derived from the intestinal tract were initially transferred into selenite F with sterile swab and incubated at 43 °C for 12–16 h. Then a loopful from selective enriched media was streaked onto plates of *Salmonella*–*Shigella* (S.S) and xylose lysine deoxycholate (XLD) agars. Loop samples of the liver and yolk sac were taken and transferred directly to plates of S.S agar and XLD agar for further incubation at 37 °C for 24 h. Tissue samples ordinarily contain relatively few competing organisms and are often transferred directly to plates of both selective and nonselective agar media, without broth enrichment (Gast 2008). Up to three suspected colonies with typical *Salmonella* morphology were confirmed biochemically by inoculating into triple sugar iron agar.

### Antimicrobial susceptibility tests

The disk diffusion method was performed to determine susceptibility of the *Salmonella* isolates based on the NCCLS 1996 protocol (National Committee for Clinical Laboratory Standards; Table 1). The bacterial suspension turbidity was adjusted to McFarland standard number 0.5 in Mueller Hinton broth (Merck) and cultured fluently over the entire surface of Muller Hinton agar with sterile cotton swab. Commercial antibiotic disks containing single concentrations of each antibiotic were then placed onto the inoculated plate surface. The zone of inhibition of growth around each disk after overnight incubation at 37 °C was measured in millimeters. The zone diameter was interpreted using a zone size interpretation chart (Lorian 1996). The antimicrobial agents tested and the corresponding concentrations were as follows: chloramphenicol 30 μg, cefotaxime 5 μg, flumequine 30 μg, colistin 10 μg, neomycin 30 μg, sulfadiazine + trimethoprim (sultrim) 15 μg, and enrofloxacin 5 μg.

**Table 1:** Antimicrobial susceptibility of *Salmonella* spp. isolated from day-old broiler chicken

Antimicrobial	Number (percent)		
	Resistant	Intermediate	Sensitive
Chloramphenicol	0 (0)	2 (3.3)	58 (96.7)
Cefotaxime	0 (0)	1 (1.66)	59 (98.33)
Colistin	13 (21.66)	5 (8.33)	42 (70)
Enrofloxacin	4 (6.66)	3 (5)	53 (88.33)
Sultrim	0 (0)	2 (3.3)	58 (96.7)
Neomycin	20 (33.33)	12 (20)	28 (46.66)
Flumecoine	7 (11.66)	3 (5)	50 (83.33)

### In vivo study

Among poultry producers, using such antibiotics during the first week of broiler management for the prevention and control of *Salmonella* and *Escherichia coli* infections is a common tradition. In the present study, enrofloxacin, sultrim, and flumequine were used in the in vivo study because of the predominant use of these three antibiotics in this age. After isolating *Salmonella* from the chickens, 40-day-old broiler chickens were purchased from related hatcheries. Chicks were randomly divided into four groups of 10 chicks per group, and antibiotics were administrated in water from day 2 for 4 days. After 10 days, samples from the liver, heart, and intestine were taken for isolation of *Salmonella*.

## Results

Out of the 730 chickens from 43 broiler breeder farms between 2008 and 2010, 60 chicks were positive for *Salmonella*. This prevalence of 8.2 % was for 12 broiler breeder farms. All 60 chicken isolates were susceptible (100 %) to chloramphenicol, cefotaxime, and sultrim. Resistance to enrofloxacin, flumequine, colistin, and neomycin were evident at 6.66, 11.66, 21.66, and 33.33 %, respectively. With regard to the in vivo study, after random sampling from the liver, heart, and intestine of chicken in each group at day 15, the following results were obtained with respect to isolating *Salmonella* spp.:

- Enrofloxacin treatment groups: growth of *Salmonella* colony was negative.
- Flumequine treatment groups: the rate of *Salmonella* colonies that grew on plates was low.
- Sultrim treatment groups: growth of *Salmonella* colonies was more than the flumequine groups.

## Discussion

Our results can be compared to the findings reported by Yildirim et al. (2011), where 90 and 97 % of isolated *Salmonella* from chicken carcasses were susceptible to chloramphenicol and cefotaxime, respectively, while just 44 % was susceptible to neomycin. These susceptibility rates are in agreement with those observed in the Zahraei Salehi et al. (2005) study. They isolated *Salmonella* from the intestine and liver of broiler chickens, and the results of the antimicrobial tests showed that all of the isolated *Salmonella* were susceptible to chloramphenicol, cefotaxime, and enrofloxacin, while 79.3 % of samples were susceptible to flumequine and trimethoprim. In one investigation performed in Portugal, poultry samples were frequently contaminated with *Salmonella* (60 %). In antimicrobial resistance tests, susceptibility to cefotaxime, chloramphenicol, and sultrim were 100, 97, and 97 %, respectively (Antunes et al. 2003). Among seven antimicrobial tests performed in Thailand, susceptibility of *Salmonella* isolated from chicken carcasses to chloramphenicol and sultrim was 100 and 98 %, respectively (Dahal et al. 2008). All of these results are in agreement with the present study.

Usage of antibiotics in poultry is for three purposes including: therapy, prevention, and growth promotion. The classes used include: β-lactams (penicillins and cephalosporins), sulfonamides β-lactams with and without trimethoprim, tetracyclines, macrolides, lincosamides and streptogramins, and quinolones (including fluoroquinolones β-lactams), which have a variety of therapeutic and preventive applications in food animals and are the same classes as those used in human therapy.

Much of the evidence relating to the potential for transfer of a resistance problem from animals to man comes from a consideration of the epidemiology of zoonoses, mainly *Salmonella* and *Campylobacter* infection (Chiu et al. 2002). The hypothesis is that the food chain is the main means of transmission. This hypothesis is intuitively attractive, and there can be no doubt of the existence of a hazard, but neither of these consideration means that the hypothesis is correct or of universal significance.

For example, enrofloxacin was not introduced for animal therapy until 1995, by which time 21 % of human isolates in one Pennsylvania study were resistant to ciprofloxacin, none having been resistant between 1982 and 1992, and by 2001, 40 % of human isolates were resistant to fluoroquinolones in this study (Nachamkin et al. 2002).

*Salmonella typhimurium* and *Salmonella infantis* are serotypes frequently isolated from food-producing animals and food poisoning cases in Japan. *S. typhimurium* DT104 and 104B were isolated from feces and diagnostic submissions mainly from cattle; some were also isolated from diagnostic submissions from swine. With the antibiotic resistance genes integrated in the chromosome, most DT104 isolates show MDR to five drugs, commonly referred to as resistance (R)-type ACSSuT (Threlfall et al. 1994). However, *S. infantis* was frequently isolated from patients suffering from food-borne illness and presents a significant public health concern related to poultry possessing strains with some resistance determinants (Esaki et al. 2003).

## Conclusion

Efforts are crucial to reduce the prevalence of resistant *Salmonella* in poultry, including the adoption of guidelines for the prudent use of antimicrobial agents in animals used for food and a reduction in the number of pathogens present on farms. The target organisms must be known or shown to be susceptible, and adequate concentrations of antibiotics must be shown to reach the target. We believe that efforts should be concentrated instead on minimizing the transmission of all food-borne pathogens regardless of their antibiotic susceptibility, by insistence on good hygiene practices on farms, in abattoirs, during distribution and marketing of food, in food preparation, and, finally, by the consumer.
